# Reproductive Capacities and Development of a Seed Bruchid Beetle, *Acanthoscelides macrophthalmus*, a Potential Host for the Mass Rearing of the Parasitoid, *Dinarmus basalis*

**DOI:** 10.1673/031.010.12901

**Published:** 2010-08-09

**Authors:** Effowe TQ, Amevoin K, Nuto Y, Mondedji D, Glitho IA

**Affiliations:** Laboratoire d'Entomologie Appliquée, Faculté des Sciences, Université de Lomé BP 1515, Lomé Togo

**Keywords:** Infestation, survival, fecundity, demographic parameters, mass rearing, biological control

## Abstract

The reproductive capacities and development of the seed beetle *Acanthoscelides macrophthalmus* (Schaeffer) (Coleoptera: Bruchidae), found in Togo, were determined under natural conditions in a Guinean zone for its use as a substitute host for the mass rearing of the parasitoid *Dinarmus basalts* Rond (Hymenoptera: Pteromalidae), a biological agent for the control of beetles that are pests of cowpea, *Vigna unguiculata* (L.) Walpers (Fabales: Fabaceae). Population dynamics at the field level; and survival, fecundity and offspring production by *A. macrophthalmus* under laboratory conditions were measured when fed on its natural plant-host *Leucaena leucocephala* (Lamark) deWit (Fabales: Mimosaceae). The data resulting from the laboratory study were used to calculate the demographic parameters of *A. macrophthalmus* by establishing its fertility and life tables. Contrary to cultivated leguminous food plants, *L. leucocephala* is a perennial sub-spontaneous leguminous plant whose pods are available year round. Although *A. macrophthalmus* was present in nature throughout the year, its infestation rate of the pods fluctuated according to the phenology of the plant. The maximum infestation of *L. leucocephala* pods was observed between August and December. Four larval stages and one pupal stage of *A. macrophthalmus* were identified in the laboratory. The total mean development time varied on average 33.75 ± 2.87 days on the mature pods and 33.39 ± 2.02 days on seeds. The adult female lived from one to two weeks. During its life time, the female laid an average of 62.3 ± 19 and 43.1 ± 13 eggs on the mature pods and seeds respectively and produced an average of 36.7 ± 11.3 offspring on the mature pods and 21.8 ± 8.4 offspring on seeds. On seeds, the net reproduction rate was 5.88 females per female and the intrinsic rate of population increase 0.051 per day. The generation time was 34.59 days and the doubling time 13.59 days. The demographic parameters of *A. macrophthalmus* in this study are close to those of *Callobruchus maculatus* F. (Bruchidae), the natural host of *D. basalts* in a previous study. *A. macrophthalmus* could therefore be used as substitute host for the mass rearing of *D. basalts* and subsequently its release in farmers' storage containers. The data presented in this study provide some baseline information regarding the reproductive capacities of *A. macrophthalmus* that may be useful for its promotion as a substitute host for mass rearing of *D. basalts*.

## Introduction

In West Africa, the Bruchidae (Coleoptera) are the main pests with great economic impact on the production of cowpea *Vigna unguiculata* (L.) Walpers (Fabales: Fabaceae), a favoured leguminous food crop for low-income rural and urban populations. The females of Bruchidae lay their eggs on developing pods of cowpea and the rate of infestation increases according to the duration of these pods in the fields (Glitho 1990). The larvae of the bruchid beetle penetrate the seeds and their entire development occurs inside. At the time of harvest, approximately 0.5% of cowpea seeds contain larvae and nymphs that will continue their development in granaries, thereby causing very significant post-harvest crop losses (Huignard et al. 1985). Three species of Bruchidae (*Callosobruchus maculatus, Callosobruchus rhodesianus* and *Bruchidius atrolineatus*) were identified in fields and storage containers of cowpea in Togo (Amevoin 1998). The latter two species had an early reproductive diapause in storage containers. Most of the cowpea damage is due to *C. maculatus* (Ouedraogo et al. 1996; Amevoin et al. 2005).

To protect their stored crops against these pests, farmers introduced into their storage structures inert substances such as ashes and sand (Chiwanda and Giga 1997), vegetable oils (Credland 1992) or presumed insecticidal aromatic plants and/or insect repellents (Golob and Webley 1980; Ketoh et al. 2006). But the effectiveness of these substances is often limited in time and applies to only small quantities of seeds (Sanon et al. 2005). In order to preserve significant quantities of cowpeas, farmers have turned to the use of synthetic insecticides, the majority of which are not intended for Bruchidae. The misuse of these insecticides inevitably has harmful consequences on the health of the users, the consumers and the environment. In addition, the widespread use of these synthetic insecticides could lead to pest resistance. To help farmers to preserve stored cowpea against beetles, it is essential to promote safe methods for the control of these pest populations. An alternative to chemical methods is the use of a biological agent to control the beetle population and consequently their damage to stored seeds (Amevoin et al. 2007). Studies of insect population dynamics on cowpea in field and granaries lead to the identification of a solitary ectoparasitoid of larvae and nymphs of Bruchidae, *Dinarmus basalis* Rond (Hymenoptera: Pteromalidae). Under natural conditions of infestation of cowpea seeds in the field, the numbers of this natural enemy are low so it does not provide an effective control of the beetle population (Amevoin et al. 2006). Biological control of the bruchids by augmentative releases of natural enemies can represent an interesting alternative for low-income small-scale producers in West Africa (Huis 1991).

Studies carried out under different experimental conditions in different climatic zones of West Africa showed that introductions of *D. basalis* adults at the beginning of storage could effectively control bruchid populations and conserve good quality of seeds after 6 months of storage (Glitho et al. 1998; Amevoin et al. 2006). Laboratory studies also showed that this parasitoid had very good discriminatory capacities of the hosts enabling it to avoid superparasitism (Gauthier et al. 1997) as well as significant parasitic effects (Sanon et al. 1998; Mondedji et al. 2002). However, the main question that always arises about the development of a biological pest control method on a large scale is the mastery of the mass rearing strategy of the parasitoid (Amevoin et al. 2006; 2007). Mass rearing tests of *D. basalis* in the laboratory require the use of significant quantities of cowpea seeds given the fact that its host, *C. maculatus*, develops within the seeds of this leguminous food. This form of mass rearing of the parasitoid consequently involves use of cowpea seeds that should normally be directly used as food by consumers. It becomes thus essential to identify species of beetles that can be developed on non-food leguminous plants and which could be used as substitute hosts for the mass rearing of *D. basalis*. A survey carried out in southern Togo (West Africa) on spontaneous and sub-spontaneous wild leguminous plants led to the identification of three Bruchidae species *Acanthoscelides macrophthalmus* (Schaeffer) (Coleoptera: Bruchidae), *Bruchidius lineatopygus*, and *Caryedon pallidus* (Bolevane 2004). Laboratory rearing tests of *D. basalis* on these three bruchid species showed that only *A. macrophthalmus* was suitable for development of this parasitoid (Bolevane et al. 2006). *A. macrophthalmus* feeds on the perennial shrub, *Leucaena leucocephala* (Lamark) deWit (Fabales: Mimosaceae) (Raghu et al. 2005; Tuda et al. 2009). However, these authors had not determined the biological characteristics of *A. macrophthalmus* that allow its development in the southern conditions of Togo. The aim of the present study is to determine the population dynamics of *A. macrophthalmus* and its life history when it is reared on its natural host plant, *L. leucocephala*. This information is essential for the use of *A. macrophthalmus* as a substitute host for rearing of *D. basalis* in a mass production unit in the south of Togo.

## Materials and methods

### Environmental conditions of the study and the experiments

The biological parameters of *A. macrophthalmus* in nature were studied in the experimental field of the University of Lomé (6° 07′N and 1° 13′E) in southern Togo in a Guinean zone. This ecological zone is characterised by two rainy seasons (April–July and September–October) separated by short and long dry seasons respectively (August and November–March). The monthly average temperatures vary from 25 to 29° C during the year and annual mean precipitations were close to 932 mm. Annual relative humidity fluctuated between 80% and 90% during the rainy season whereas it varied from 67% to76% in the dry season. Rearing of *A. macrophthalmus* and the study of its reproductive capacities and development were carried out in ambient conditions (28 ± 2° C, 73 ± 3% RH) in the laboratory. The photoperiod is approximately 12:12 L:D in southern Togo.

### Study of population dynamics of *A. macrophthalmus* in nature

Population dynamics of *A. macrophthalmus* was followed in the approximately 1 hectare *L. leucocephala* plantation located in the western part of the University of Lomé campus and exploited as fodder plant. Weekly random harvests of 100 ripe pods of *L. leucocephala* on 20 different plants were carried out throughout the year in 2005. The pods collected were observed in the laboratory and those from which eggs (fresh or old) of *A. macrophthalmus* were laid were counted. From the counts, the rate of natural infestation of the pods by the wild bruchid was determined. The population dynamics of *A. macrophthalmus* in the pods harvested was estimated by collecting and counting the adult beetles emerging every 2 days from these pods during 49 days (the maximum of development time of this species in order to avoid the overlapping of two generations). Parallel to the study on population dynamics of *A. macrophthalmus*, observations and follow-up of the phenology of *L. leucocephala* were carried out every week. The relationship between the phenology of the plant and the population dynamics of this insect was then determined.

### Evaluation of the reproductive capacities and development of *A. macrophthalmus*

The adults of *A. macrophthalmus* emerging from the pods collected in nature were reared in the laboratory on seeds of *L. leucocephala* allowed sufficient individuals of *A. macrophthalmus* to be obtained for the experiments. The reproductive capacity and the development cycle of *A. macrophthalmus* was evaluated on two substrates, ripe pods or seeds of *L. leucocephala*. Before starting any experiment, these substrates were placed in a freezer for two weeks in order to eliminate any form of former infestation. When the adults of *A. macrophthalmus* resulting from the rearing conditions emerged, 2 batches (A and B) of 50 pairs consisting of a male and female were formed. Each pair of batch A was placed in a box (10×3×4 cm) containing 5 ripe pods and each pair of batch B was placed in a similar box containing 50 seeds (approximately equivalent to the number of seeds in 5 pods). The pods and the seeds of each couple from each batch were renewed daily until the death of the female adults. The pods and the seeds from each batch were then observed daily until the emergence of the next generation of adults. The number of eggs laid daily by each female in each batch was counted. The following parameters were recorded at the end of the experiment:

- the lifespan of the females of each batch;
- the number of eggs laid per day and per female;
- the date of formation of the first larva in the egg (neonatal larva);
- the total number of adults (male and female) emerged;

All these parameters were used to determine:

- the survival rate of the female adults;
- the infestation rate of the pods and seeds;
- the mean fecundity corresponding to the number of eggs laid by female during its lifetime as well as the daily variations of eggs laid;
- the fertility rate represented by the number of eggs in embryonic stage compared to the total number of eggs laid by female;
- the emergence rate represented by the number of emerged offspring compared to the total number of eggs laid by female;
- the sexual ratio (% of females) and the durations of development of the male and female offspring.

The parameters measured only on seeds were used to establish the fertility and life tables of *A. macrophthalmus* according to Morales-Ramos and Cate (1992), and the Doury and Rojas-Rousse (1994) method applied for *D. basalis* by Mondedji et al. (2002). From these tables, growth parameters [net reproduction rate (R_0_); generation time (G); intrinsic rate of population increase (r_m_) and doubling times of the population (DT)] of *A. macrophthalmus* reared on seeds under Guinean zone conditions in southern Togo were calculated using the same calculations as that of the authors cited above.

In order to identify the different stages of the immature *A. macrophthalmus* and to determine their duration, 40 batches of 50 seeds of *L. leucocephala* each with one or two eggs were made. The seeds with eggs were obtained from a daily mass rearing of *A. macrophthalmus* in a large box (7×28×29 cm). Daily dissections of one of the 40 batches were made starting from the penetration of the first larval stage in the seed and continuing until the emergence of the adults. The number of larvae and/or nymphs was recorded and their development stages were determined by counting the number of exuviae present in the seed. Their development durations were given starting from the date of the eggs were laid to the date of the dissection of the batches.

### Statistical analyses

The average number of the different parameters of the experiments was calculated. The comparisons of the mean numbers were made using analysis of variance (ANOVA-1) followed by Student-Newman-Keuls (SNK) comparison tests when *F* of analysis of variance was significant at the 5% level. Emergence rate were compared with χ^2^ test. The results were analysed using the software Statistica 5.1, edition 1998.

**Figure 1.**
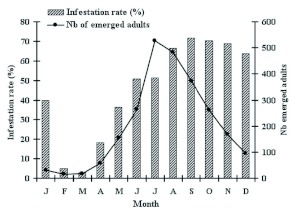
Infestation rate and emergece of adults of *Acanthoscelides macrophthalmus* on pods of *Leucaena leucocephala* in 2005. High quality figures are available online.

## Results

### Temporal variation of *A. macrophthalmus* density in nature

In the laboratory, observation of the pods collected in the field showed that *A. macrophthalmus* is present in nature all year round (Figure 1). However, the rate of infestation of the *L. leucocephala* pods in nature varied according to the month. Flowering and the fructification of *L. leucocephala* occurred throughout the year. The pods were consequently present throughout the year but the ripe pods were abundant in June, August and December. The pods remained for a longer time on the *L. leucocephala* tree if they were still indehiscent. In this condition, the beetle laid its eggs throughout the year. However, the females preferred to deposit their eggs on the new ripe pods. The rates of infestation of the pods and the number of emerged adults were low during the second half of the long dry season between February and March, corresponding to the period when the majority of the *L. leucocephala* trees partially lose their foliage and when there are still old ripe and dry pods from the last season. These old pods had innumerable emergence holes of *A. macrophthalmus* adults and did not seem to be chosen by the females for oviposition. The infestation rates of the pods increased in April and a maximum rate of attacks occurred between August and December (64 and 72% of the pods were attacked). This period corresponds to the good flowering and fructification season of the host plant and hence with the availability of the new pods that are preferentially selected by females for oviposition. In the same way, the number of emerged adults increased and reached a peak in July before starting to decrease up to January.

### Life history parameters of *A. macrophthalmus* adults Age-dependent adult female survival

**Figure 2.**
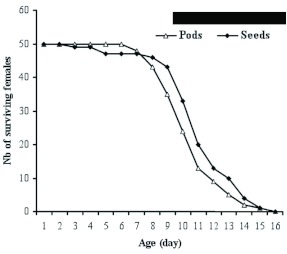
Age-dependent female (n = 50) survival of *Acanthoscelides macrophthalmus* reared on pods and seeds of *Leucaena leucocephala*. High quality figures are available online.

The mean longevity of *A. macrophthalmus* adult females was 10.5 ± 1.9 and 11 ± 2.5 days respectively on pods and seeds but this was not significant (F = 1.27; df = 1; P = 0.2631). The survival of the females was thus not a function of the nature of the substrate (seed or pod) on which they deposited their eggs. Survival was high when the age of the female was between 1 and 7 days (Figure 2) independently of the nature of the substrate that the female deposited her eggs on. From the 8^th^ day, the number of surviving females decreased significantly until the 16^th^ day when all of the females had died.

### Age-dependent fecundity

**Figure 3.**
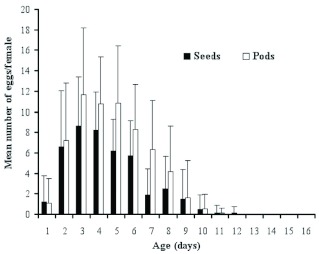
Age-dependent fecundity by *Acanthoscelides macrophthalmus* reared on pods and seeds of *Leucaena leucocephala*. High quality figures are available online.

A female laid an average of 62.4 ± 19 eggs on pods and 43.1 ± 13.8 eggs on seeds during its life (Table 1). The difference was statistically significant (F = 34.29; df = 1; P < 0.0001). Age-dependent fecundity of *A. macrophthalmus* was divided into three periods (Figure 3): the pre-ovipositional period (from the emergence of the adult female to the first oviposition) was estimated at 1 day on pods or seeds; the oviposition period lasted 7.5 ± 1.5 days on pods and 6.5 ± 2.2 days on seeds (F = 7.48; df = 1; P < 0.0074); and the post-oviposition period (from the last oviposition to the death of the female) varied from 2.1 ± 1.4 on pods to 3.4 ± 1.9 days on seeds (F = 14.09; df = 1; P < 0.0003). The number of eggs laid by *A. macrophthalmus* during the oviposition period fluctuated according to the substrate of the oviposition and the age of the females (Figure 3). The females deposited the maximum number of eggs (6 to 12 eggs) between the 2^nd^ and the 6^th^ days with a peak on the 3^rd^ day after their emergence (a mean number of 11.6 ± 6 and 8.7 ± 4.7 eggs respectively on ripe pods and seeds). From the 7^th^ to the 12^th^ day after emergence, fecundity was low.

### Development and survival of immature stages

The average duration of egg development was 4.52 ± 0.84 days with a fertility rate of 65.7% (Table 2). Four larval (L_1_, L_2_, L_3_ and L_4_) and one pupal stages were identified. These stages developed inside the seed. Mean larval development lasted 4.2, 4.7, 5.3, 5.2 and 5.7 days for larvae and pupae stages respectively. The longest durations were recorded at the stages L3, L4 and pupa. Survival rates were 73% at the L_1_ stage, 91% at the L_2_ and L_3_ stages and 86% at the pupal stage. Survival rates of larvae and pupae were higher than that of eggs. Total development (egg to adult) lasted 33.8 ± 2.9 days on pods and 33.4 ± 2 days on seeds (F = 4.26; df = 1; P < 0.039). These means were significantly different according to the substrate of oviposition: it was longer on pods than on seeds (Table 3).

### Offspring production and sex-ratio

Emergence of adults spread from the 26^th^ to 42^nd^ day after oviposition on the seeds and from the 28^th^ to 44^th^ day after oviposition on the pods. During their lifetime, the females produced an average 36.7 ± 11.3 offspring, consisting of 18.6 ± 6.4 females and 17.8 ± 6.5 males (sex-ratio of approximately 50.30% females) when the eggs were laid on pods (Table 1). On seeds, the females produced an average 21.8 ± 8.4 offspring, which corresponded to 11.1 ± 4.8 females and 10.6 ± 4.76 males. The sex-ratio on seeds was 50.9%. The mean number of offspring were significantly different according to the substrate but the sex-ratios were practically identical (Table 1). The emergence rate was 58.5% on ripe pods and 50.6% on seeds (the difference was not statistically significant, χ^2^c = 0.634; df = 1).

### Demographic parameters

Demographic parameters were deduced from data on fertility and survival (Tables 4 and 5). Survival probability (L_x_) decreased according to the age classes (Table 4). It fluctuated from 1 to 0.60 for the immature stages and from 0.52 to 0.19 for the adult stages. Consequently, the life expectancy (Ex) was high at the level of the immature stages and became low in age groups 2 and 3, corresponding to the last age classes of the adult.

The mean number of female progeny per female (m_x_) varied according to the age class. In the first age class (which lasted 1 to 5 days after emergence of the female progenitor), the number of female progeny per female was 8.60 (Table 5). This value decreased as the age class increased. The reproductive value (V_x_) of the females was higher in the first age class and it decreased in the others. Summary of the growth parameters is given in Table 6. The net reproduction rate (R_0_) of *A. macrophthalmus* was 5.88 days. The generation time (G) was equivalent to 34.36 days. The intrinsic rate of population increase (r_m_) was 0.051 and the doubling time of the population (DT) 13.59 days.

**Table 1.**
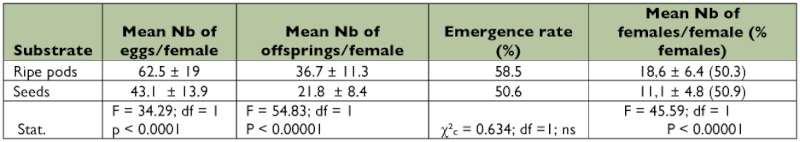
Reproductive potential of the females of *Acanthoscelides macrophthalmus* (n = 50 females) according to the substrate.

**Table 2.**
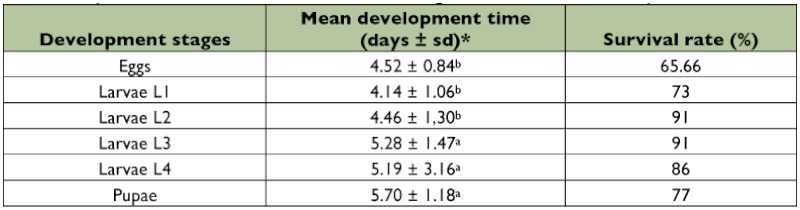
Mean development time and survival rate of immature stages of *Acanthoscelides macrophthalmus*.

**Table 3.**
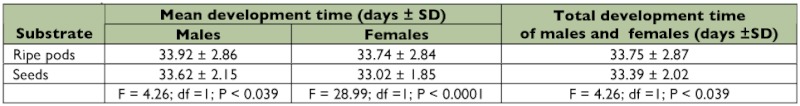
Total development of males and females of *Acanthoscelides macrophthalmus* reared on ripe pods and seeds of *Leucaena leucocephala*.

## Discussion

Under the natural conditions of our study (a one-year survey in nature in the latitude of Lomé, Togo), *A. macrophthalmus* was found to be the only bruchid that developed in *L. leucocephala*, an introduced plant in Togo. This perennial plant was introduced in 1980 to promote its use as fodder, as firewood and for atmospheric nitrogen fixing by international forestry organizations (Sue and Kobie 2004). Research carried out in Togo did not record *A. macrophthalmus* as a bruchid that develops in cultivated leguminous plants (Glitho 1990). Moreover in its areas of origin, *A. macrophthalmus* was listed as a bruchid that attacked several wild leguminous plant species all belonging to *Leucaena* genera (Elder 2002). These observations enable us to affirm that *A. macrophthalmus* does not develop on cultivated leguminous plants, which is a good reason to consider it as a substitute host for the rearing of *D. basalis*.

In nature, the females of *A. macrophthalmus* deposited their eggs on the pericarp of the ripe pods. Several generations of larvae can develop in the same pod because these pods remain on the *L. leucocephala* tree for a long time. This observation was also confirmed for bruchids reproducing on cultivated species of cowpea and for some wild bruchids (Amevoin 1994; Raghu et al. 2005). In addition to chorions of old eggs , the pods collected throughout the year carried the fresh eggs of *A. macrophthalmus*, suggesting that this insect was reproductively active throughout the year round contrary to some beetle species that reproduce in cultivated cowpea and which have a reproductive diapause (Huignard et al. 1985; Amevoin et al. 2005). In the laboratory, the experiments showed that *A. macrophthalmus* laid eggs on pods as well as on seeds. Nevertheless, the fecundity of the females differs significantly when the eggs were laid on ripe pods compared to when laid on seeds of *L. leucocephala*. The female of *A. macrophthalmus* like those of many species of Bruchidae, controls its fecundity according to the nature of the substrate for oviposition. It has been shown that in contact with various oviposition substrates, females of phytophagous insects perceived with their tactile and gustatory receptors the difference in the texture and chemical composition of the plant-host (Huignard 1984; Parr et al. 1996). The signals coming from the usual and natural host (ripe pods) appears to stimulate increased oviposition, as has been observed in the bean beetle *Acanthoscelides obtectus* Say (Pouzat 1981; Monge 1983). However, the signals leading to the choice of the oviposition substrate by the female seem in general to be complex. The fecundity of *A. macrophthalmus* on ripe pods is close to that of *A. obtectus* (Parson and Credland 2003) but differs from that of the Togolese flightless morph stream of *C. maculatus*, which laid an average 86.5 ± 6.60 eggs on cowpea pods (Glitho 1990).

**Table 4.**
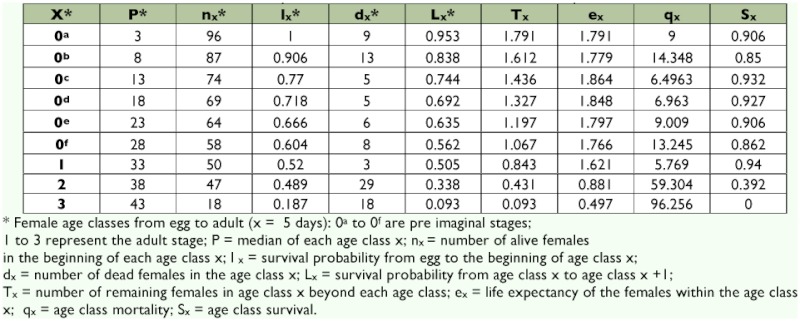
Life table of *Acanthoscelides macrophthalmus* reared on seeds of *Leucaena leucocephala*.

**Table 5.**
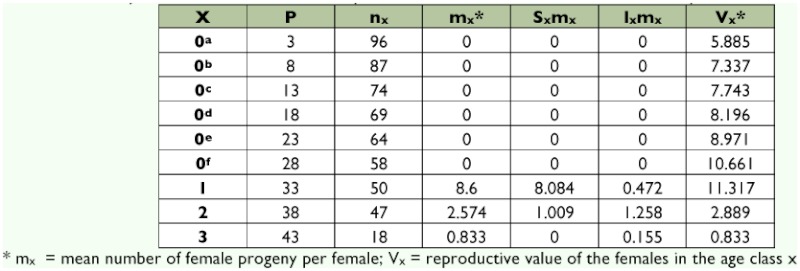
Fertility table *of Acanthoscelides macrophthalmus* reared on seeds of *Leucaena leucocephala*.

The age-dependent fecundity of the females showed that *A. macrophthalmus* has a pre-oviposition period and lays very few eggs at the beginning of its imaginai life. This suggests that when the females eclose, their ovarioles are not yet functional. Oogenesis apparently takes place gradually after the emergence of the adult females. The significant variations in the mean number of eggs laid per female during different ages is an indication of great individual variability in fecundity as observed in many species of insects (Amevoin 1998). The results obtained in nature and in the laboratory showed that *A. macrophthalmus* laid on pods and seeds of *L. leucocephala* throughout the year which is a second reason for it being a potential substitution host for the rearing of *D. basalis*. Based only on the fecundity and development of *A. macrophthalmus* on the two substrates in the laboratory, the study showed that the ripe pods are the best substrate for rearing of the beetle. Nevertheless, pods are very cumbersome and their handling is very difficult in the laboratory. In addition, they require much space, incompatible with mass rearing. However, the experiments also showed that seeds were used for oviposition in the laboratory and can be used in the mass rearing of bruchids.

**Table 6.**
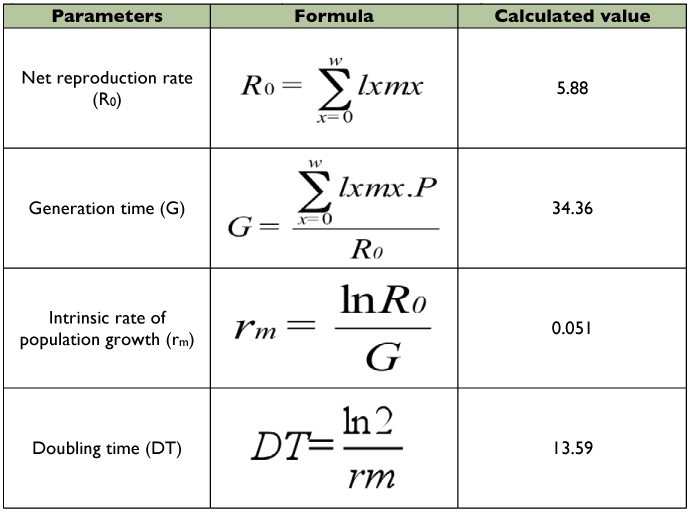
Growth parameters of *Acanthoscelides macrophthalmus* in the laboratory.

During the development of *A. macrophthalmus* in laboratory, the survival rate varied according to immature stages. It was low at the first larva stages (L1) probably because of the extreme hardness of the tegument of *L. leucocephala* that can constitute a barrier for the penetration of the first larva instars inside the seed. At the pupal stage, mortality was also significant that may be related to the morphological and physiological disruption during metamorphosis (Mondedji et al. 2002). The survival rate recorded in immature stages was certainly the cause of the reduction in the survival (Lx) and life expectancy (Ex) at these stages of development. The significance of the various barriers the first larva stage must cross when the development takes place on the pods (pericarp of the pod, seed tegument) is the main reason that explains the long total development of *A. macrophthalmus* when the eggs are laid on pods as compared to when they are laid on seeds. This behaviour was also observed in various other species of bruchids (Huignard et al. 1985). As observed in *C. maculatus, A. macrophthalmus* can also develop successfully in laboratory conditions but its total duration of development is slightly longer than on the natural hosts of *D. basalis*.

The evolution of a population can be predicted by the analysis of fertility and survival tables (Dajoz 1974). According to Doury and Rojas-Rousse (1994), the intrinsic rate of population increase, r_m_ is of great importance because its calculation provides a means to compare the reproductive potential of the species under various biotic and abiotic conditions. It can also be used to compare several species under the same conditions. The determination of the demographic parameters of *A. macrophthalmus* on seeds (the less cumbersome substrate) allowed a comparison between the reproductive capacity of this beetle and that of *C. maculatus*, the natural host for development of the parasitoid *D. basalis*. The reproductive value (V_x_) of *A. macrophthalmus* showed that the females of the first age class (1–5 days after emergence of adult females) produced more female progenies than that of the females of the other age classes. Any rearing of *A. macrophthalmus* for mass production of *D. basalis* should necessarily take into account this age class factor. The estimated growth parameters of *A. macrophthalmus* are close to those reported by Ndoutoume (1996) for *C. maculatus* under control conditions similar to those of our climatic zone, which leads to the third rationale for the importance of this wild bruchid for mass rearing of the parasitoid *D. basalis*.

The data presented in this study provide some baseline information regarding the reproductive capacities of *A. macrophthalmus* that may be useful for its promotion as a substitute host for mass rearing of *D. basalis*. Nevertheless, some additional studies on the adaptation of the new streams of *D. basalis* reared on *A. macrophthalmus* to the natural host *C. maculatus* are warranted before any promotion of this wild bruchid as a substitute host for the parasitoid.

## Abbreviations

R_0_: net reproduction rate;
G: generation time;
r_m_: intrinsic rate of population increase;
DT: doubling time
